# Latest Advances in Inhalable Dry Powder Bacteriophage Therapy for Pulmonary Infections

**DOI:** 10.3390/pharmaceutics17081077

**Published:** 2025-08-20

**Authors:** David Encinas-Basurto, Patricia Dolores Martinez-Flores, Joselyn García, Marco Antonio Lopez-Mata, Gerardo García-González, Gerardo E. Rodea, Basanth Babu Eedara, Heidi M. Mansour, Josue Juarez

**Affiliations:** 1 Centro de Investigación en Alimentación y Desarrollo, A.C. Carretera a La Victoria Km. 0.6, Hermosillo 83304, Sonora, Mexico; 2 Departamento de Física, Posgrado en Nanotecnología, Unidad Regional Centro, Universidad de Sonora, Hermosillo 83000, Sonora, Mexico; a204200368@unison.mx (P.D.M.-F.); a220230145@unison.mx (J.G.); 3 Departamento de Ciencias de la Salud, Universidad de Sonora, Campus Cajeme, Blvd. Bordo Nuevo S/N, Antiguo Ejido Providencia, Ciudad Obregón C.P. 85010, Sonora, Mexico; marco.lopezmata@unison.mx; 4 Departamento de Microbiología, Facultad de Medicina Y Hospital Universitario “Dr. José Eleuterio González”, Universidad Autónoma de Nuevo León, Monterrey 64460, Nuevo Leon, Mexico; gerardo.garciagnzl@uanl.edu.mx; 5 Laboratorio de Investigación en Microbiología y Resistencia Bacteriana, Hospital Infantil de México Federico Gómez, Márquez 162 Col. Doctores, Alcaldía Cuauhtémoc, Mexico City 06720, Mexico; ge_rodm@hotmail.com; 6 Center for Translational Science, Florida International University, Port St. Lucie, FL 34987, USA; babubasanth@gmail.com (B.B.E.); hmansour@fiu.edu (H.M.M.)

**Keywords:** bacteriophage therapy, inhalable powders, nanoparticles, liposomes, multidrug-resistant pathogens

## Abstract

The concerning increase in respiratory infections that are resistant to multiple drugs has led to a growing interest in bacteriophage therapy as a potential alternative to conventional antibiotics. Effective phage delivery to the lungs, however, presents several formulation and stability issues, particularly for inhalation-based methods. This review highlights current developments in the creation of dry powder formulations that can be inhaled for pulmonary phage therapy, with a focus on encapsulation methods based on nanoparticles, such as solid lipid nanoparticles (SLNs) and polymer-based nanoparticles. These carriers enhance the aerodynamic characteristics of phages, making them suitable for deep lung deposition, while also protecting them during processing and storage. Several drying methods have been investigated to create powders with optimal morphologies, porosity, and dispersibility, including spray drying and spray freeze drying. The review also emphasizes how the phage morphotype affects stability, especially when nebulization stress is present. Furthermore, the advantages of nanoparticle matrices are confirmed by the reduced viability loss (usually< 0.5 log PFU) of encapsulated phages. Standardizing production processes, scaling up, and ensuring regulatory compliance remain challenging despite encouraging preclinical results. The combination of phage therapy with nanotechnology creates new avenues for the utilization of inhalable delivery methods to treat multidrug-resistant pulmonary infections. To translate these novel formulations from preclinical development to clinical application, sustained multidisciplinary collaboration across pharmaceutical sciences, microbiology, and clinical pharmacology is essential.

## 1. Introduction

Multidrug-resistant (MDR) bacterial infections present a serious global health threat, particularly in chronic pulmonary diseases such as bronchiectasis, ventilator-associated pneumonia, chronic obstructive pulmonary disease (COPD), or cystic fibrosis (CF) [1]. Patients with these conditions frequently experience recurrent infections that are challenging to manage due to bacterial resistance mechanisms, including reduced drug uptake, target site modification, enzymatic drug inactivation, and the active efflux of antimicrobial agents. In addition, biofilm formation significantly increases antimicrobial resistance and compromises the efficacy of conventional inhaled therapies.

**Figure 1:**
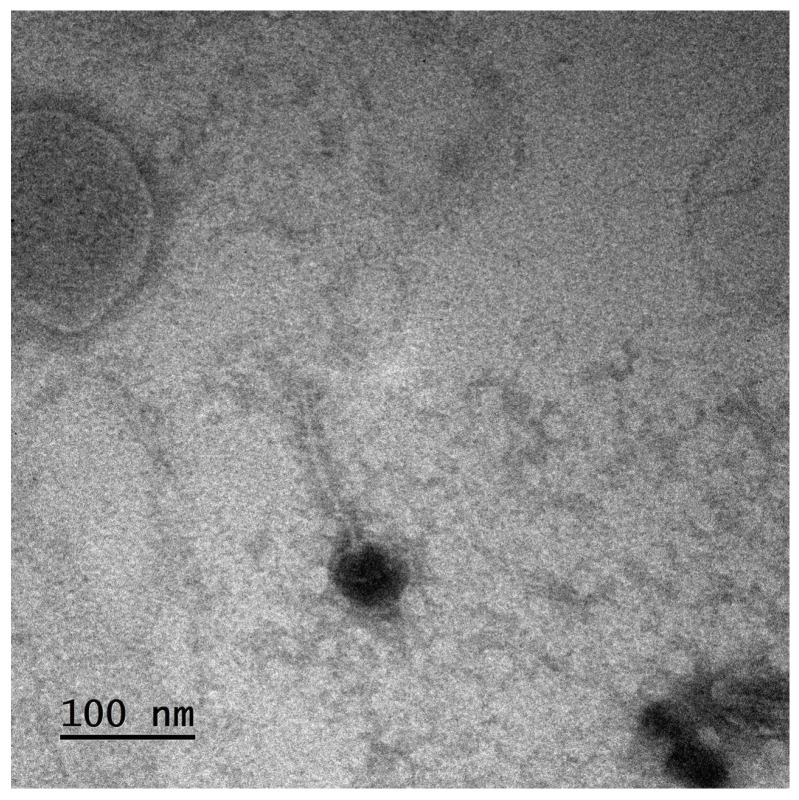
Morphological characterization of bacteriophage vB_Eco_T01 by transmission electron microscopy (TEM).

Bacteriophages, or phages, are viruses that exclusively infect and replicate within bacteria. Derived from the Greek word “*phagein*”, meaning “to devour”, this term reflects their capacity to destroy a specific bacterium. Phages are composed of genetic material (DNA or RNA) encapsulated within a protein shell called a capsid and exhibit diverse sizes and morphologies. They are the most abundant biological entities in the biosphere, with an estimated 10^31^ particles globally, and play a vital role in microbial ecology and bacterial population dynamics [2] (Figure 1).

Phage therapy has reemerged as a promising treatment for chronic lung infections, particularly those exacerbated by MDR pathogens. These include bronchiectasis, COPD, and diseases related to CF. The therapeutic advantages of phages include their high specificity toward bacterial targets, ability to replicate at the infection site, and adaptability to host bacteria. Traditionally, phage therapy has been administered in liquid form, often via nebulization. However, concerns regarding phage stability during storage and efficient delivery to deep lung regions have prompted the development of dry powder inhalation (DPI) formulations [3]. Dry powder formulation offers numerous benefits, including enhanced stability, an optimized particle size for deep lung penetration, and increased patient compliance due to its portability and ease of use [4,5]. These features position DPI formulations as a highly promising alternative for the treatment of persistent pulmonary infections.

**Figure 2:**
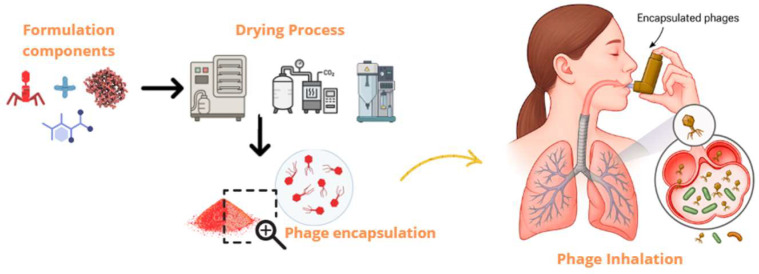
Phages are combined with excipients and processed using drying techniques such as spray drying, freeze drying, or supercritical CO_2_ drying to produce dry powders. These powders encapsulate phages and are administered via DPI, enabling targeted delivery to the lungs for the treatment of respiratory infections caused by multidrug-resistant bacteria.

In addition, designing inhalable bacteriophage formulations requires a thorough understanding of powder engineering. This field enables the optimization of critical physicochemical features, such as particle size, shape, bulk density, and hygroscopicity, all of which play crucial roles in ensuring successful lower airway deposition. Using techniques such as spray drying, freeze drying, and upcoming technologies like supercritical fluid spray drying, respirable particles can be generated to protect phage viability during processing, storage, and administration (Figure 2). As a result, powder engineering is positioned as a critical pillar in transforming this therapeutic strategy into practical clinical applications. This review examines the latest advancements in lung infection treatment using inhalable dry powder bacteriophage therapy. It discusses the significance of inhalation phage therapy, the limitations of present inhaled therapies, the need for dry powder formulations, and the importance of such formulations in improving stability, bioavailability, and delivery effectiveness.

## 2. Bacteriophages as a Therapy for Pulmonary Infections

### 2.1. Mechanism of Action of Lytic Phages in Targeting Pseudomonas aeruginosa

**Figure 3:**
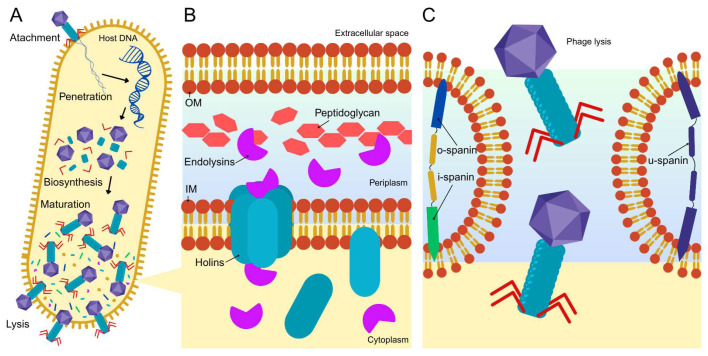
Mechanisms of bacteriophage-induced bacterial cell lysis. (**A**) The lytic replication cycle of bacteriophages begins with the binding of the phage to a specific receptor on the surface of a prokaryotic cell, followed by the injection of viral DNA. This is succeeded by the transcription and translation of viral proteins, the assembly of new virions, and, ultimately, the lysis of the host cell. (**B**) The molecular mechanism involves the expression of viral genes encoding holins, which form pores in the inner membrane. These pores facilitate the translocation of endolysins into the periplasm, where they degrade the peptidoglycan layer. (**C**) Spanins contribute to the final stage of lysis by promoting membrane fusion, thereby enabling the formation of pores through which mature phage particles are released. Spanins may contain protein domains with affinity for the inner membrane (i-spanin), the outer membrane (o-spanin), or both (u-spanin).

Phage therapy refers to the use of bacteriophages to eliminate bacterial pathogens. Although phages are generally regarded as highly specific, their host range is determined by both structural and genetic features that enable bacterial recognition and infection, as well as by viral characteristics that influence bacterial recognition and infection. Phages typically follow one of two replication cycles: the lytic cycle or the lysogenic cycle. The lytic cycle is of therapeutic relevance [6]. In this pathway, after the phage recognizes and binds to the bacterial surface receptor, it injects its genetic material into the host cell. The phage then commandeers the host’s replication machinery to produce progeny virions, culminating in bacterial cell lysis (Figure 3).

The most well-characterized lytic mechanism involves the holin–endolysin system, where two classes of phage-encoded proteins coordinate bacterial cell wall degradation. Holins are small transmembrane proteins that accumulate in the bacterial inner membrane and, at a critical concentration, form pores. These pores allow the release of endolysins—muralytic enzymes—into the periplasm, where they hydrolyze the peptidoglycan layer, comprising structural integrity [7]. In Gram-negative bacteria like *P. aeruginosa*, an additional outer membrane prevents complete lysis from peptidoglycan degradation alone. Many phages therefore encode a third group of proteins known as spanins, which mediate the fusion of the inner and outer membranes, resulting in complete lysis and phage release [8]. Spanins may exist as a biocomponent system—an inner membrane (i-spanin) and an outer membrane (o-spanin)—or a single unified u-spanin that spans both membranes [9]. Lytic phages such as PAK_P1, KTN4, and LUZ24 targeting *P. aeruginosa* have been shown to encode these essential components. Structural and mutational studies have demonstrated that the loss of any of these components impairs complete lysis, highlighting their important role in the phage lytic cycle [7].

Beyond the holin–endolysin–spanin triad, some phages, particularly giant phages like phiKZ or EL, employ alternative lytic strategies. This may involve interference with lipopolysaccharide synthesis or novel protein complexes that bypass classical lysis pathways [10]. The elucidation of these mechanisms has enabled the design of engineered lytic enzymes, or enzybiotics, which serve as standalone antimicrobial agents. Recombinant endolysins have demonstrated high efficacy against Gram-positive bacteria through chemical conjugation with membrane-permeabilizing peptides [11]. Holins have also attracted interest in synthetic biology as programmable triggers for cell lysis. Spanins, while less studied, hold promise in enhancing lytic activity in therapeutic formulations.

Overall, lytic phages targeting *P. aeruginosa* possess evolutionarily optimized systems to overcome the structural defenses of Gram-negative bacteria. Understanding these mechanisms is vital in advancing phage-based interventions and developing novel antimicrobials.

### 2.2. Advantages over Inhaled Antibiotics

*P. aeruginosa* airway infections are frequently associated with recurrent hospitalization, often due to the failure of community-based treatments and their contribution to progressive airway damage [12]. This resistance to treatment is primarily attributed to bacterial adaptive mechanisms that promote survival against conventional antibiotics. One of the most significant is the formation of a biofilm, a structural barrier composed of bacterial aggregates embedded within a self-produced extracellular matrix, primarily consisting of alginate and extracellular DNA. This matrix not only impedes antibiotic penetration but also increases bacterial tolerance up to 1000-fold compared to planktonic cells [13].

Biofilm-resident bacteria display heterogeneous metabolic activity: cells near the surface are more active and accessible, whereas those located deeper within the biofilm adopt a slow metabolic state, which confers increased tolerance to antimicrobials [14]. Consequently, intravenous antibiotic therapies often require high dosages that carry risks of systemic toxicity and facilitate the emergence of resistant strains, ultimately contributing to chronic infection [15]. In contrast, inhaled antibiotics offer significant advantages, including localized drug delivery, reduced systemic side effects, and improved patient adherence due to ease of use [16]. Delivery platforms such as nebulizers, metered-dose inhalers (MDIs), and DPI have been widely explored [17].

Despite these advantages, current inhaled antibiotic formulations often exhibit limited efficacy against mature *P. aeruginosa* biofilms. Initially, treatments typically reduce planktonic bacteria and associated inflammation, creating an illusion of clinical improvement, while the underlying biofilm persists [18]. For instance, Høiby et al. [19] demonstrated that tobramycin inhibited planktonic and early biofilm-forming cells but failed to eradicate mature biofilms, as confirmed via agar diffusion assays and confocal microscopy.

In response, advanced therapeutic systems have been developed. One promising approach involves co-loaded dry powders combining antibiotics such as ciprofloxacin and colistin within liposomal carriers. Using ultrasonic spray freeze drying, these formulations produce particles (4.3 µm diameter) with an over 99% emitted dose and show potent antibiofilm activity by efficiently diffusing through mucus and neutralizing bacterial toxins. In mouse models, these systems achieved a 99.7% reduction in lung bacterial colonization, which was significantly attenuated [20,21].

Combination therapies, often supplemented with mucolytic agents or biofilm dispersants, further enhance antibiotic penetration and activity. Agents such as clindamycin, tigecycline, tobramycin, gentamicin, and imipenem have demonstrated improved efficacy against biofilm-embedded *P. aeruginosa* when administered in combination [22]. Other innovative strategies include pairing antibiotics with natural antibiofilm and immunomodulatory compounds or using phage–antibiotic combinations.

Notably, dry powder co-delivery systems exploiting bacterial metabolic responses have shown promise. For example, glutamic acid, a metabolically attractive nutrient, can stimulate biofilm dispersion by drawing dormant bacteria to more superficial layers, rendering them more susceptible to ciprofloxacin. This approach reduced the required antibiotic dose by approximately 20% [23]. Similarly, quercetin combined with ciprofloxacin and mucolytics such as mannitol and N-acetylcysteine in a spray-dried formulation achieved a high emitted dose and demonstrated potent antibiofilm activity [24].

**Figure 4:**
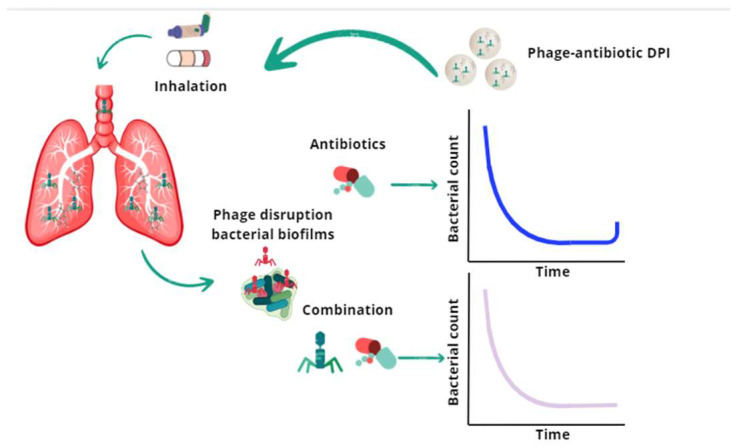
Inhaled phage–antibiotic DPI enhances bacterial clearance in the lungs. The combination disrupts biofilms and improves antibiotic efficacy, leading to greater bacterial reduction over time.

Conversely, other authors have explored the synergistic potential of phage–antibiotics as alternative antibiofilm agents, inhibiting the growth of bacteria (Figure 4). Phage therapy represents a further advancement, offering high specificity, self-replication, and efficacy against biofilms, with minimal host toxicity. Synergistic phage–antibiotic combinations have been shown to reduce the necessary antibiotic dose while maintaining or enhancing the therapeutic efficacy [25]. For instance, phage PEV20 co-administered with ciprofloxacin or gentamicin in inhalable dry powder or nebulized formulations demonstrated synergistic bactericidal effects and reduced biofilms associated with *P. aeruginosa* [6,12].

In preclinical studies, the intratracheal delivery of PEV20–ciprofloxacin powders to mice resulted in a 5.9-log reduction in the bacterial burden within 24 h, outperforming monotherapies. Moreover, these powders exhibited favorable aerosolization profiles with fine particle fractions exceeding 70% [25]. Although clinical validation is ongoing, these findings highlight the substantial therapeutic potential of phage–antibiotic dry powder combinations in managing MDR *P. aeruginosa* infections.

A recent study by Hong et al. [26] has shown that bacteriophages and antibiotics interact effectively to improve the effectiveness of antimicrobial therapy, especially when it comes to *Pseudomonas aeruginosa*, which is resistant to many drugs. In one study, the lytic phage PEV20 was examined in both planktonic and biofilm settings with ciprofloxacin and colistin. The phage–ciprofloxacin combination produced a 4-log drop in the number of bacteria in planktonic cultures when compared to controls, indicating a clear synergistic effect, according to the results. Colistin considerably outperformed each drug alone in biofilm models, reducing viable bacteria by more than 2.5 logarithmic units. The potential of this strategy in persistent infections was further supported by time-kill experiments, which demonstrated that co-treatment not only improved bacterial clearance but also delayed regrowth. Significantly, cytotoxicity tests conducted on A549 lung epithelial cells showed that the combination therapies were well tolerated, highlighting their appropriateness for delivery by inhalation. These results demonstrate the potential of phage–antibiotic synergy as a focused strategy to enhance treatment results in respiratory infections brought about by resistant Gram-negative bacteria.

### 2.3. Phages’ Ability to Evolve and Adapt Against MDR Bacteria

Phages possess evolutionary and molecular strategies that enable them to infect and lyse MDR bacteria, disrupt biofilms, and evade bacterial defense mechanisms, all while minimizing harm to eukaryotic cell hosts [27]. In parallel, bacteria have developed a diverse arsenal of defense mechanisms to resist phage infection, resulting in an ongoing co-evolutionary arms race between these microbial adversaries [28].

A key bacterial defense strategy involves altering or downregulating surface receptors that phages use for adsorption. Such changes, often derived by spontaneous mutations, reduce the bacterial susceptibility to infection [3]. In response, phages can evolve by acquiring mutations in genes encoding tail-fiber or baseplate proteins, which are responsible for binding to receptors. These adaptive mutations restore infectivity and can expand, allowing phages to target resistant strains within a matter of days or weeks [28,29].

Efflux pumps constitute another bacterial defense mechanism. These transport proteins expel harmful substances, including antibiotics and potentially phage components, from the intracellular environment. Interestingly, some phages exploit components of these efflux systems, such as TolC in *Escherichia coli* or MexAB-OprM in Pseudomonas, as entry points. If bacteria mutate these components to block phage entry, they often compromise pump efficiency. This adaptive trade-off renders the bacteria more susceptible to antibiotics, a phenomenon referred to as partial resensitization [30,31,32].

Restriction–modification (R-M) systems are also widely used by bacteria to degrade foreign DNA. These systems consist of restriction endonucleases (REases), which recognize and cleave specific DNA sequences, and methyltransferases (MTases), which methylate host DNA to prevent self-cleavage [33]. To overcome these defenses, phages produce anti-restriction proteins, such as the Ocr protein from phage T7, which mimics DNA and inhibits REase activity [34]. Additional counterstrategies include encoding decoy proteins or chemically modifying phage genomes to mimic the patterns of host DNA.

Another bacterial defense is the Clustered Regularly Interspaced Short Palindromic Repeats (CRISPR-Cas) system. This adaptative immune mechanism captures short sequences from invaders and uses them to guide nucleases for the targeted degradation of foreign DNA. In response, many phages have evolved anti-CRISPR (Acr) proteins, which block all stages of the CRISPR-Cas pathway. To date, over 120 distinct Acr proteins have been identified. These proteins block crRNA-guided recognition, Cas nuclease activation, or nucleic acid cleavage. Some Acr proteins even degrade bacterial signaling molecules, acting through unique and complex biochemical pathways [35,36,37].

Phages also possess enzymes known as exopolysaccharide depolymerases, which degrade the biofilm matrix and facilitate phage penetration. Phage cocktails containing depolymerase-producing strains have been shown to significantly reduce biofilms formed on medical devices and tissues [38,39].

Recent studies have encountered a growing number of novel bacterial defense systems, suggesting that the co-evolutionary interaction between phages and bacteria is even more intricate than previously understood. Examples of newly discovered systems include the cyclic oligonucleotide-based anti-phage signaling system (CBASS), Pycsar and Thoeris, Gabija and Hachiman, Zorya, BREX, DISARM, and Wadjet and Septu [40]. These systems rely on complex intracellular signaling networks, nucleic acid modifications, and protein effectors to detect and neutralize phage threats, highlighting the remarkable diversity of bacterial immune responses.

Overall, the dynamic interplay between phages and bacterial defense systems underscores the immense evolutionary plasticity of phages. Their ability to adapt rapidly and counteract even the most complex bacterial strategies reinforces their therapeutic potential and paves the way for the development of innovative antimicrobial approaches.

## 3. Dry Powder Inhaler Systems for Bacteriophage Therapy

DPI systems have emerged as a promising platform for pulmonary bacteriophage delivery due to their portability, stability, and ability to deliver high therapeutic loads directly to the lungs [41]. Compared to nebulizers or pressurized metered dose inhalers (pMDIs), DPI systems offer several advantages, including rapid drug administration, improved stability, and the ability to deliver high concentrations of therapeutic agents directly to the lungs [42].

Bacteriophage DPI systems are generally classified into three categories: carrier-based, carrier-free, and nanoparticle-embedded systems (Table 1) [43]. Carrier-based DPI systems use excipients to enhance powder flow and dispersion but may dilute the phage payload. Carrier-free formulations eliminate such excipients to increase the aerodynamic performance and therapeutic potency. Nanoparticle-embedded systems, including polymeric and lipid-based carriers, enhance protection, enable controlled release, and improve the phage delivery efficiency [44,45].

Carrier-based DPI systems represent the most straightforward approach and typically utilize excipients such as lactose or mannitol to improve powder flow and dispersibility. However, the inclusion of carriers may dilute the phage payload and reduce the therapeutic potency [46]. Stabilizers and dispersibility enhancers are critical in DPI formulation. While lactose has been proven to be an effective excipient in DPI formulations by enhancing phage stability and dispersity, its primary drawbacks include its involvement in Maillard reactions and its unsuitability for individuals with lactose intolerance [47]. In contrast, non-reducing sugars not only avoid these issues but also contribute to protein stabilization in the solid state by forming an amorphous matrix that immobilizes proteins in their native conformation and by replacing water molecules through hydrogen bonding with protein structures. The amorphous glass provides more physical and chemical stability than the amorphous rubbery state, so it is essential to have a high glass transition temperature, T_g_, that is well above room temperature. The amorphous rubbery state has higher molecular mobility and a greater void volume than the amorphous glass; hence, water vapor can be absorbed into the amorphous rubber, causing plasticization, leading to physical and chemical instability.

Leucine aids aerosol dispersion by reducing particle cohesion. These excipients are typically selected based on their compatibility with phage structures and their ability to preserve bioactivity under drying and storage conditions. Formulations combining lactose and leucine have shown fine particle fractions (FPFs) of up to 45% and extended stability for up to 12 months [48].

**Table 1:** Comparative overview of DPI systems for phage delivery.

DPI Type	Phage	Technology	Advantages	Limitations	Reference
Carrier-based	PEV1, PEV20, PEV61 against *P. aeruginosa*	Lactose + leucine, spray dried	Stable up to 12 months, non-toxic to lung cells in vitro	Requires controlled-humidity storage	[41]
Carrier-free	Phage LUZ19 and Romulus	Spray drying with 4% trehalose solution	Forms respirable particles (1–5 µm)	Crystallization at high RH reduces viability	[49]
Nanoparticle-embedded	*K. pneumoniae* phage cocktail	Nanostructured lipid carrier	Mechanical protection, extended therapeutic effect	More complex formulation	[50]

The main challenge in developing carrier-free formulations lies in minimizing particle cohesion while maximizing the dispersion and delivery efficiency [51]. These systems allow for higher drug or phage payloads per dose, thereby reducing the total powder mass required for therapeutic efficacy. Additionally, carrier-free DPI formulations can be tailored to address specific patient needs and disease profiles, offering greater flexibility in personalized medicine approaches.

Enhanced phage deposition in the target lung region can be achieved by precisely tuning particle characteristics, such as the size, density, morphology, and surface composition, through formulation and process optimization [52,53].

Conversely, the encapsulation of bacteriophages in nanoparticles offers multiple benefits, including protection from environmental stress, prolonged stability, and controlled release [54] (Table 2). These systems act as both protective carriers and delivery platforms, maintaining phage titers at therapeutic levels. To prevent phage inactivation during drying, encapsulation within micro- and nanoparticles has gained considerable attention. Spray drying and freeze drying are the primary techniques used to produce phage-loaded dry microparticles. Although these methods enhance the stability, handling, and shelf life, they may also compromise phage activity. To mitigate this, phages can be encapsulated in liposomes, polymeric carriers, or nanohydrogels [55].

Agarwal et al. [61] developed PLGA-based polymeric particles loading phages targeting *P. aeruginosa*, with an initial particle size of approximately 8 µm. When co-formulated with lactose into a DPI system, the aerodynamic diameter was optimized to 1–5 µm, enabling efficient deep lung deposition. In murine models, inhalation of these formulations led to a significant reduction in bacterial load, demonstrating high phage encapsulation efficiency, room-temperature stability, effective pulmonary release, and the evasion of alveolar macrophage clearance. Additionally, liposome-based dry powder formulations have emerged as promising candidates for pulmonary administration. These systems can maintain stability during storage and delivery and retain biological activity following processes such as nebulization or spray drying. Southard, Melton, Sandoval, Zaki, Williams III, and Cui [60] demonstrated that liposome-encapsulated bacteriophages represent a promising strategy for the treatment of respiratory infections via inhalation, offering advantages such as enhanced protection against environmental degradation and improved lung deposition.

### 3.1. Drying Techniques for DPI Phage Formulations

Spray drying involves atomizing a liquid feed containing phages into fine droplets, which are subjected to rapid water evaporation under controlled high-temperature conditions. Key process variables include the atomization pressure, feed rate, viscosity, surface tension, inlet/outlet temperatures, drying gas flow, and resident time. These parameters influence the particle morphology, moisture content, and phage viability [62]. As indicated in Table 3, the structural sensitivity of the phage type and its pulmonary use should be taken into consideration when choosing a drying strategy, in addition to process efficiency.

Each drying technique used to formulate phage-based dry powders presents distinct advantages and limitations that influence the formulation performance, production cost, and phage viability. Among these, spray drying is the most commonly adopted method due to its rapid processing, scalability, and compatibility with existing pharmaceutical manufacturing infrastructure. However, the elevated inlet temperatures typically used (100–170 °C) can comprise the structural integrity of thermosensitive phages unless protective excipients are used [66]. Despite this limitation, spray drying remains a cost-effective approach and consistently yields particles with favorable aerodynamic characteristics.

In contrast, lyophilization (freeze drying) is conducted under low-temperature conditions, preserving phage infectivity. Nevertheless, it often results in large, irregularly shaped particles that require downstream milling to attain respirable sizes. This additional processing introduces mechanical stress and significantly increases the energy consumption and processing time, reducing the commercial feasibility of lyophilized products for high-yield dry powder inhalers [67]. Spray freeze drying (SFD) has emerged as a promising low-temperature alternative that produces highly porous, low-density particles with superior dispersibility while preserving structural integrity. SDF techniques have demonstrated phage recovery rates exceeding 85%, especially for heat-sensitive strains. However, these methods are associated with higher production costs, extended processing times, and limited scalability [68].

Finally, TFF is an innovative technology for the production of brittle matrix particles characterized by low thermal stress and efficient aerosolization. Despite its promise, TFF remains underexplored for phage formulations, with limited data on long-term stability and viability, particularly under GMP-complaint, large-scale manufacturing conditions [69]. For large-scale manufacturing, spray drying is still the most viable method, particularly when combined with excipients to reduce heat stress. Even though they are more expensive, methods like atmospheric spray freeze drying or SFD might be beneficial for phages that are structurally sensitive or in scenarios where maximal viability is crucial. Therefore, depending on the particular application and phage type, the decision should balance biological preservation, aerodynamic performance, production efficiency, and regulatory compliance.

**Figure 5:**
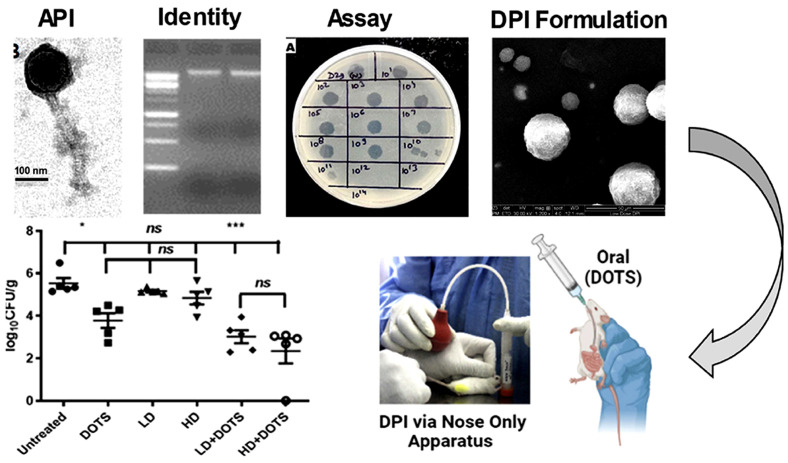
Workflow of DPI-based bacteriophage therapy characterization. The figure illustrates key steps including phage identity confirmation (TEM and electrophoresis), functional assays (spot test), DPI powder formulation (SEM), and the evaluation of therapeutic efficacy in vivo using both oral (DOTS) and intranasal (nose-only inhalation) delivery in a mouse model. The graph shows the lung bacterial burden (log_10_ CFU/g) across various treatment groups, highlighting the superior effects of phage–DPI formulations. Adapted from [64].

In a recent study by Raman, Roy, Verma, Yadav, Verma, Deivreddy, Sofi, Bharti, Sharma, Bansode, Kumar, Sharma, Singh, Mugale, Bajpai, Jain, Singh, and Misra [64], dry powder inhalers containing mycobacteriophages D29 and TM4 were evaluated in a murine model of *Mycobacterium tuberculosis* infection. The phage-based DPI therapy demonstrated comparable, if not superior, efficacy to a conventional oral anti-tuberculosis treatment with isoniazid and rifampicin (Figure 5). After four weeks of treatment, oral human-equivalent doses of isoniazid and rifampicin reduced pulmonary Mtb colony-forming units (CFU) from 6.4 ± 0.3 log to 4.8 ± 0.7 log. In contrast, daily inhalation of the phage-based DPI formulation, containing around 10^10^ plaque-forming units/dose, achieved a greater reduction to 3.8 ± 0.8-log. Notably, a combination therapy involving both oral ATT and inhaled phages further reduced the group mean CFU to 2.3 log, with the complete sterilization of the lungs observed in one of the four treated mice.

Raman, Roy, Verma, Yadav, Verma, Deivreddy, Sofi, Bharti, Sharma, Bansode, Kumar, Sharma, Singh, Mugale, Bajpai, Jain, Singh, and Misra [64] demonstrated that the rapid dissolution of dry powder formulations in lung pulmonary surfactant facilitates the immediate release of free-floating phages upon inhalation. This contrasts with encapsulation strategies, such as liposomes, alginate beads, or other nano-/microformulations, which retain phages post-delivery and rely on gradual release. However, neither approach has shown a clear enhancement in phage bioavailability at the primary site of infection. Extracellular Mtb may persist in biofilm-like structures or within poorly vascularized granulomas, complicating therapeutic access. To enable phage access to phagosome lumen, specialized mechanisms such as endosomal fusion or engineered scape strategies are required. While nano- and microcarriers may enter via endocytosis, effective intracellular delivery remains a major barrier.

In a recent study, Southard, Melton, Sandoval, Zaki, Williams III, and Cui [60] demonstrated that TFF is an effective technique for the development of inhalable dry powder formulations of bacteriophages targeting multidrug-resistant *Pseudomonas aeruginosa* and *Klebsiella pneumoniae*. These formulations exhibited excellent aerodynamic properties (MMAD of 2.5–3.2 µm, FPF > 60%) and high phage viability (80–90%) and retained lytic activity following ambient storage. The study emphasized the importance of incorporating stabilizing excipients to ensure powder stability and dispersibility.

### 3.2. Comparative Performance of DPI vs. Nebulized Formulations

Dry powder formulations for bacteriophage delivery offer numerous advantages over traditional nebulized systems. DPI formulations provide enhanced stability due to their solid-state nature, protecting phages from mechanical shear, enzymatic degradation, and environmental stress during storage and administration. In contrast, nebulized phages, especially in liquid suspensions, are more susceptible to inactivation during aerosolization. Studies demonstrate that DPI formulations exhibit reduced phage titer losses compared to nebulized systems. For example, the viability losses in nebulized Myoviridae phages ranged from 0.65 to 1.2 log PFU, while Podoviridae showed slightly lower reductions (0.3–0.8 log PFU). Conversely, DPI-manufactured phages retained up to 90% viability under optimized drying and formulation conditions [70].

While most phage research has focused on treating respiratory infections using liquid aerosols administered via intranasal instillation, the development of inhalable phage powder formulations has emerged as a promising alternative. These powder systems not only enhance phage stability and shelf life but are also user-friendly and improve patient compliance [71,72]. Table 4 summarizes representative studies that have employed spray-drying and freeze-drying techniques for phage powder development for pulmonary delivery.

**Figure 6:**
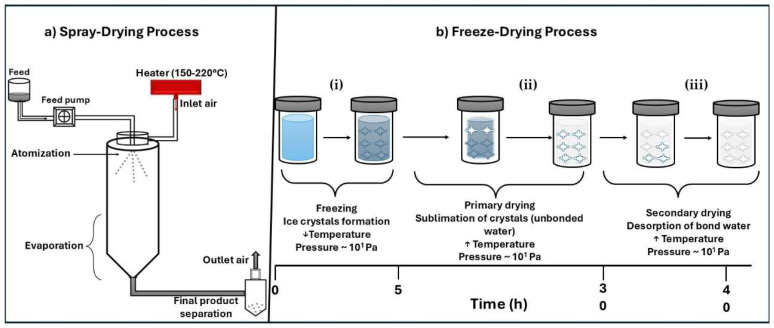
Schematic representation of (**a**) spray-drying and (**b**) freeze-drying. In freeze-drying process involves three sequential stages: (i) freezing, where ice crystals form; (ii) primary drying (15–20 h), during which unbound water is removed by sublimation; and (iii) secondary drying, in which bound water is desorbed to yield the final powder.

Ergin [74] compared spray drying, freeze drying, and electrospraying phage-loaded carriers using whey protein, inulin, gum arabic, and Tween 80. Electrosprayed powders had the highest bulk and tapped densities, while freeze drying preserved the highest phage activity, followed by spray drying [17]. Figure 6 presents a schematic representation of spray-drying and freeze-drying techniques.

## 4. Key Characteristics of Inhalable Phage Powders

In carrier-based DPI systems, the particle size plays a critical role in the aerosolization efficiency. Spray drying has proven effective in achieving the optimal aerodynamic diameter (typically 0.5 to 5 μm), enhancing the success of deposition in the alveolar region. Particles larger than 5 μm tend to deposit in the oropharyngeal region, limiting their efficacy for deep lung delivery. Conversely, excessive size reduction can introduce challenges due to the increased surface area, which intensifies interparticle cohesion and adhesion. This adversely affects the powder flow properties and complicates the formulation [44,75]. Due to their high surface-area-to-volume ratios, inhalable particles often exhibit poor flowability and strong cohesive tendencies. Carrier particles are typically incorporated to enhance power flow, facilitate device filling, and ensure consistent dose delivery. During inhalation, drug particles must efficiently detach from carrier surfaces, and agglomerates must disperse in the airstream [75].

Since bacteriophages are sensitive to environmental factors and mechanical stress during aerosolization, ensuring resistance to humidity and mechanical degradation is vital in DPI development. Excipients such as trehalose can form glassy, amorphous matrices that shield phages during spray drying and storage [45]. For instance, trehalose has been shown to preserve the infectivity of MS2 phages across a wide relative humidity (RH) range of 20–80%. In contrast, phages with long tails, such as Φ6, require low RH of 20% to avoid structural damage [76]. These advances are essential in improving phage viability and therapeutic efficacy in the treatment of resistant pulmonary infections.

## 5. Stability and Viability of Dry Powder Phages

**Figure 7:**
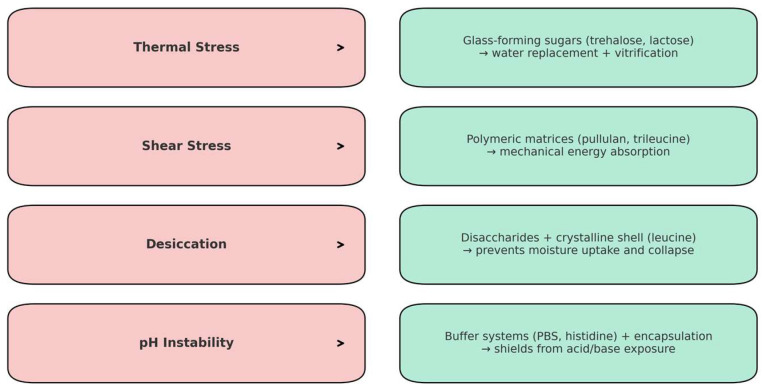
Nanoformulation strategies aligned with key phage degradation pathways commonly encountered during dry powder formulation.

The development of DPI for inhalable bacteriophage therapy offers a promising avenue by which to address MDR pulmonary infections. However, ensuring the long-term stability and biological activity of phages during production, storage, and pulmonary delivery remains a significant challenge (Figure 7).

Glass-forming sugars such as lactose, sucrose, or trehalose are among the most effective stabilizers for the preservation of phages during the drying process. These disaccharides function by replacing structural water in phage particles through hydrogen bonding, forming a protective amorphous matrix that immobilizes the phages in a dry, inert state. This vitrified matrix prevents protein unfolding and aggregation, preserving structural integrity and infectivity. Trehalose, for instance, has demonstrated superior stabilizing properties due to its ability to sustain a glass transition temperature (Tg) above typical storage conditions. This high Tg reduces molecular mobility and moisture uptake, limiting degradation. Several investigations have reported minimal phage potency loss, typically less than 0.5 log units, when trehalose is used as the primary excipient [44,63]. In addition to sugars, excipients such as leucine and trileucine are used as shell-forming agents to improve powder dispersibility. These amino acids form a crystalline shell around the particles, enhancing the aerosol performance and providing mechanical protection against shear stress, moisture, and oxidation during storage and delivery [77]. This section discusses the key physicochemical and biological factors that impact the viability of dry powder phage formulations.

### 5.1. Mechanical Stress During Drying Process

Bacteriophages are inherently sensitive to the physical stresses encountered during drying processes, which can compromise their structural integrity and infectivity. Spray drying, a commonly used technique to produce inhalable powders, subjects phages to thermal and shear stresses during atomization and rapid solvent evaporation. These conditions can result in the denaturation of capsid proteins or the irreversible aggregation of viral particles. Similarly, lyophilization exposes phages to stress associated with ice crystallization, solute concentration gradients, and interfacial phenomena (e.g., ice–air and ice–water interfaces), which may destabilize phage particles and reduce their viability [78].

The high shear forces generated during nozzle atomization in spray drying can damage critical phage structures, such as capsid proteins and tail fibers. Studies on lactic acid bacteria have demonstrated similar shear-induced inactivation mechanisms, with viability losses linked to the nozzle pressure and feed viscosity. The inlet temperatures during spray drying (typically 60–80 °C) may also induce heat shock, leading to protein denaturation and genomic damage. Although brief, such exposure can significantly reduce infectivity, particularly if protective strategies are not implemented.

The incorporation of excipients, particularly amino acids such as L-isoleucine, has been proven to be effective in mitigating heat stress during drying. Recent studies have demonstrated that phages targeting Salmonella retain infectivity after exposure to 80 °C, provided that the formulation includes optimal concentrations of L-isoleucine. This amino acid creates a protective matrix that preserves capsid integrity and mitigates heat-induced denaturation [79]. These stabilizers act via water replacement, vitrification, and protein structure stabilization. For instance, trehalose has a high glass transition temperature (Tg ~115 °C), allowing it to immobilize phage particles within an amorphous matrix and limit conformational changes during drying and storage [11,80]. Sugars such as trehalose and sucrose form hydrogen bonds with the polar groups of viral proteins, replacing the water–protein hydrogen bond interactions that are typically formed by water molecules and thereby preserving the native conformation of the proteins. This water replacement mechanism is essential to avoid denaturation and loss of phage infectivity during dehydration [81,82].

In agreement with recent mechanistic studies, bacteriophages can be successfully incorporated into polymer-based carriers or matrices, such as casein, PLGA, or pullulan, to mitigate inactivation caused by shear forces during atomization in dry process such as spray drying. These carriers help to redistribute phages away from the particle surface by absorbing kinetic energy [83]. Notably, combinations such as trehalose and casein or trileucine have been associated with enhanced long-term stability at both ambient and frozen storage conditions, as well as reduced titer losses following spray drying. Furthermore, although mannitol alone may cause protein destabilization through post-drying crystallization, its incorporation in small amounts with amorphous sugars has been shown to improve storage performance. Formulations containing 30–40% mannitol have demonstrated reduced water sorption and particle aggregation, provided that crystallization is effectively delayed or inhibited [68,84].

Experimental studies have shown that phage inactivation during spray drying is primarily driven by capsid destabilization and protein denaturation, particularly in the absence of protective excipients [85]. Formulations lacking disaccharides such as lactose or trehalose exhibited complete inactivation, with titer reductions exceeding 6–8 log_10_ units. Conversely, lactose combined with leucine demonstrated superior protective effects in PEV phages, showing less than 1-log titer loss post-drying and yielding particles < 3 μm, ideal for lung deposition; see Chang, Wong, Mathai, Morales, Kutter, Britton, Li, and Chan [85].

Freeze drying also presents risks during freezing, as ice crystals can physically disrupt phage structures, and solute exclusion creates highly concentrated microenvironments that are potentially denaturing. Interfacial stresses (i.e., ice–liquid, solid–vacuum) further promote capsid aggregation and destabilization [57,86]. Zheng [87] reported that sucrose-based formulations maintained high phage viability during freeze drying and remained stable for over 1000 days at 4 °C, due to their amorphous structures. In contrast, formulations with glucose or mannitol showed significant infectivity losses, attributed to crystallization. This underscores the importance of selecting excipients that not only protect during lyophilization but also prevent devitrification under real-world storage conditions.

### 5.2. Humidity Effects on Long-Term Storage

Relative humidity (RH) is a critical factor affecting the long-term stability of dry powder phage formulations. At a low RH level (0–20%), formulations containing trehalose or lactose retain a stable amorphous matrix that immobilizes phage particles and protects them from degradation. Consequently, phage viability is well maintained for months at 4 °C [77,84]. Polymers included in the formulation can further limit water uptake, acting as moisture barriers.

However, as the RH increases above 50–60%, it can lead to significant moisture absorption, which plasticizes the matrix, lowers the glass temperature (Tg), and induces recrystallization in sugar-based systems. In polymer-based systems, high RH can cause increased chain mobility, phase separation, and the loss of matrix integrity, depending on the polymer composition and hygroscopicity [63,66].

Polymers are often used to stabilize phages but show varied effectiveness. Excipients such alginate, agar, pullulan, PEG, and polyvinylpyrrolidone (PVP) form matrices that increase the Tg, immobilize phages, and offer protection [78]. For example, alginate hydrogels improve phage stability and allow controllable release [88]. Pullulan combined with trehalose significantly enhanced long-term stability in dried films compared to trehalose alone [89]. Despite these advantages, some polymers, such as PEG and PVP, have been shown to be incompatible with specific phages. For instance, PEG 6000 and PVP have been linked to immediate titer loss post-lyophilization or during storage, likely due to steric hindrance or unfavorable protein interactions [90].

Most inhalable phage powders rely on disaccharides, amino acids, and polyols due to their regulatory approval and compatibility with spray drying or lyophilization. However, polymer encapsulation, using materials such as chitosan, alginate, or poly(lactic-co-glycolic acid) (PLGA), offers potential for enhanced protection and controllable pulmonary release. These systems remain underexplored due to challenges in aerodynamic optimization, reduced bioavailability, and the limited regulatory precedent for inhaled polymers. Future research should investigate polymer-based dry powder systems as alternatives or complements to sugar-based formulations. Their potential to provide moisture protection, mechanical stability, and controlled release may enable better long-term viability and the functional retention of inhaled bacteriophages.

### 5.3. Thermal-Mediated Phage Instability

Thermal stress is a key degradation mechanism for phages during formulation, particularly in spray drying, where temperatures can exceed 100 °C. Although direct exposure is short, these high temperatures can destabilize viral capsids, denature structural proteins, and accelerate nucleic acid degradation. These effects are frequently initiated by the partial denaturation of capsid proteins, which exposes hydrophobic regions that promote aggregation and functional loss. Notably, temperature tolerance varies among phage families, with Myoviridae phages typically demonstrating greater resilience than Podoviridae or Siphoviridae under identical processing conditions [91].

To reduce these adverse effects, nanoformulation strategies incorporate excipients with well-established thermoprotective properties [44,63]. Trehalose can substitute for structural water at the capsid junction, forming hydrogen bonding that preserves the protein conformation. Leung et al. (2017) [77] demonstrated that incorporating trehalose and leucine improved phage stability and powder dispersibility in spray-dried formulations. While mannitol can enhance matrix rigidity, it tends to crystallize during drying, which may compromise phage recovery unless paired with amorphous excipients [84]. Despite these promising approaches, there remains a lack of direct comparative studies evaluating the responses of different phage families to specific thermal profiles and excipient systems. Such data would support rational formulation design based on the phage morphology and processing history. Furthermore, while trehalose-based systems have demonstrated excellent efficacy, developing materials such as pullulan, silk fibroin, and thermally stable synthetic polymers might open up new pathways for the stabilization of thermosensitive phages. As dry powder inhalation technologies approach clinical trials, understanding the thermodynamics of phage–excipient interactions will be critical in developing robust and scalable pharmaceuticals.

### 5.4. pH-Mediated Phage Instability

Phages are particularly susceptible to pH-induced degradation during formulation or after pulmonary delivery, especially when exposed to non-physiological environments. Most phages maintain structural stability within a narrow pH range of 6.0 to 8.0 [44]. Beyond this range, both capsid proteins and nucleic acids can undergo chemical or conformational destabilization. In acidic environments, the protonation of ionizable amino acid residues, such as glutamate, aspartate, and histidine, can disrupt the electrostatic interactions essential for capsid integrity. This frequently leads to premature DNA ejection, capsid expansion, or the dissociation of the tail fibers. Additionally, acid hydrolysis can accelerate phosphodiester bond cleavage and depurination, irreversibly damaging the phage genome [92].

To prevent these effects, buffering agents such as histidine or phosphate-buffered saline are incorporated into the feed solution during spray drying by nanoformulations. These buffers help to maintain a stable microenvironment around the phage during dehydration and rehydration, minimizing pH changes [77]. Encapsulation within pH-neutral or protective polymeric matrices, such as chitosan, PLGA, or alginate, further protects phages from direct exposure to external acidic or basic conditions. For example, multilayered chitosan–alginate systems have demonstrated enhanced bioactivity and phage retention in simulated environments with varying pH levels, such as those found in the pulmonary and gastrointestinal systems.

Liposomal delivery systems also contribute to pH stabilization [93]. Lipid bilayers composed of phospholipids can inhibit proton penetration, protecting encapsulated phages in acidic aerosols or inflammatory tissue microenvironments. For instance, Salmonella-targeting phages encapsulated in liposomes exhibited improved resistance to inactivation in simulated gastric fluids compared to non-encapsulated controls. Additionally, pH-responsive polymers such as Eudragit S100 have also been investigated for targeted drug release in specific pH zones [94,95].

While liposomes or pH-sensitive carriers regulate release profiles and protect against rapid acidic exposure, buffers maintain the local pH during processing, and encapsulating materials such as chitosan or PLGA offer mechanical and biological separation. Together, these strategies support the therapeutic use of phages in complex gastrointestinal and pulmonary settings, greatly improving their survivability under pH-related stress. To improve stability and controlled release, Jamaledin et al. [96] demonstrated that filamentous phages, designed as nanovaccines, can be successfully encapsulated within PLGA microparticles. PLGA offers a protective microenvironment against pH variations and chemical stress, according to their research. They observed that, by changing the polymer molecular weight and lactic–glycolic acid ratio, which alters the internal micro-pH, the rates of PLGA degradation and phage release can be changed. Since infection or inflammation can cause local pH fluctuations, tunability is essential for pulmonary delivery. Their study concluded that employing PLGA formulations increases phage bioavailability while preserving functionality in acidic environments.

## 6. Stress Conditions for Bacteriophages During Delivery System Formulation Process

The development of bacteriophage-based therapies targeting *Pseudomonas aeruginosa* requires the careful consideration of how formulation processes affect phage viability and therapeutic performance. These processes often impose physical and chemical stressors, such as thermal exposure, shear forces, dehydration, and pH fluctuations, that may comprise phage stability. For example, phage PEV20, a member of the Myoviridae family, retained its viability after undergoing spray-drying processes for the preparation of inhalable dry powders, both in formulations with ciprofloxacin and as a monotherapy (Table 5). In these formulations, the use of excipients preserved phage stability even after aerosolization, while also demonstrating synergistic effects with antibiotics against clinical multidrug-resistant *P. aeruginosa* strains [6].

In preclinical models, the activity of dry powder PEV20 remained stable under moderate relative humidity, and its intrapulmonary administration in mice with acute lung infections significantly reduced the bacterial load without evident loss of activity due to formulation stress [97]. Furthermore, liquid formulations of phages such as PEV1 and PEV61, from the Myovirus and Podovirus genera, respectively, also demonstrated good tolerance to nebulization using conventional devices, without significant loss of bactericidal activity, positioning them as viable candidates for inhaled therapies [98]. While liquid formulations may be less stable over time, they offer practical advantages in terms of immediate availability and ease of administration.

**Table 5:** Phages stress responses under delivery formulations.

Common Name of Phage	Scientific Name of Phage (Family)	Experimental Model	Formulation Type	Excipient Used	Effect on Phage Activity	Reference
PEV20	Myoviridae	In vivo murine model with *P. aeruginosa*	Dry powder for inhalation	L-leucine and lactose	No activity loss, synergistic with ciprofloxacin	[6]
PEV20	Myoviridae	In vivo murine model with *P. aeruginosa*	Dry powder for inhalation	L-leucine	No activity loss, synergistic with ciprofloxacin	[6]
PEV20	Myoviridae	In vivo neutropenic mouse model	Dry powder for inhalation	L-leucine	5.9 × 10^10^ CFU reduction, synergistic with ciprofloxacin	[97]
phiYY	Cystoviridae	In vivo clinical model of cystic fibrosis	Nebulization	Liquid solution	Stability limited by gastric pH	[99]
PEV1	Podoviridae	In vivo murine lung infection model with *P. aeruginosa*	Spray-dried powder	Trehalose or lactose and leucine	<1 log_10_ loss with trehalose/lactose + leucine; without sugar or leucine, up to 8 log_10_ loss	[85]
PEV20	Myoviridae	In vivo murine lung infection model with *P. aeruginosa*	Spray-dried powder	Trehalose or lactose and leucine	Similar to PEV1; lactose + leucine most effective (0.4–0.9 log_10_ loss)	[85]
PEV61	Podoviridae	In vivo murine lung infection model with *P. aeruginosa*	Spray-dried powder	Trehalose or lactose and leucine	Similar to PEV1 and PEV20; best with lactose + leucine (0.3–0.4 log_10_ loss)	[85]
Phage 95 (ATCC 14211-B1)	Not specified (likely Podoviridae)	In vitro H441 lung epithelial model	Liposomal inhalable formulation	Hydrogenated phosphatidylcholine, cholesterol, DSPE-PEG, mannitol, sucrose	Viability improved; 0.64 log reduction after nebulization; 2-fold lower cellular uptake	[100]
PEV31	Podoviridae	In vivo, BALB/c mice	Intratracheal suspension	Phosphate-buffered saline + 1 mM CaCl_2_	Maintained infectivity initially; gradual decline in absence of bacteria; proliferation in presence of *P. aeruginosa*; slight inflammatory response at high dose	[101]
PEV2	Podoviridae	In vitro aerosol model using Osmohaler	Spray-dried powder	Trehalose, mannitol, L-leucine	~1.5 log loss overall; ~80% phage loss during aerosolization but higher total lung dose	[102]
PEV2	Podoviridae	In vitro aerosol model using Osmohaler	Spray freeze drying	Trehalose, mannitol, L-leucine	~2 log loss during atomization; ~20% phage loss during aerosolization, lower lung dose	[102]
LUZ19	Podoviridae	In vitro	Spray-dried powder	D-trehalose + L-isoleucine	High concentration of L-isoleucine improves stability; activity loss < 1 × 10^10^ pfu/mg	[103]
14-1	Myoviridae	In vitro	Spray-dried powder	D-trehalose + L-isoleucine	High concentration of L-isoleucine reduces activity; activity loss < 1 × 10^10^ pfu/mg	[103]

Nevertheless, not all phages respond equally to formulation stress. For instance, encapsulated phages such as phiYY, from the Cystoviridae family, exhibit sensitivity to acidic environments such as the stomach, thereby limiting their use in oral formulations unless properly protected [99]. Similarly, phages like MPK6 and PNM, classified within the Phikmvvirus genus, show environmental sensitivity to temperature and pH when used in liquid solutions, necessitating stringent storage and handling protocols to preserve their bioactivity [104]. These observations highlight the need to tailor formulation strategies to the physicochemical properties and inherent stability of each phage, as well as to the excipients employed.

## 7. Pulmonary Immune Clearance Mechanisms for Phages

**Figure 8:**
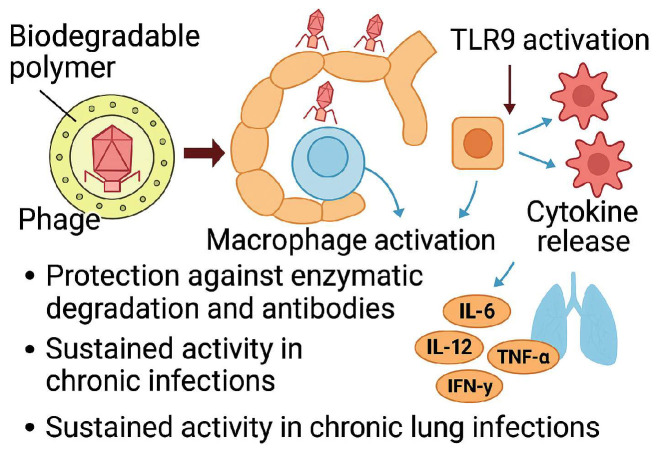
Encapsulation of phages in biodegradable polymers protects them from degradation and enhances sustained activity. Phage uptake by macrophages activates TLR9 and induces cytokine release (IL-6, IL-12, TNF-α, IFN-γ), supporting the immune response during chronic pulmonary infections.

Once inhaled, bacteriophage particles encounter multiple mechanical and immunological barriers within the respiratory system, which can significantly impact their therapeutic efficacy (Figure 8). These barriers include mucociliary transport, enzymatic degradation, alveolar macrophage phagocytosis, and the activation of both innate and adaptive immune responses [105,106].

The first barrier is mucociliary clearance, a mechanical defense system that traps inhaled particles in mucus and transfers them upward toward the pharynx via coordinated ciliary beating. While this mechanism effectively clears particles larger than ~5 µm, smaller particles may also be affected if they are mucoadhesive. The rapid clearance of phage-containing dry powder formulations can limit their residence times in the lungs, potentially resulting in subtherapeutic phage concentrations unless strategies are used to enhance mucosal adherence and retention [107].

In the alveolar region, alveolar macrophages act as sentinel phagocytes capable of rapidly internalizing phage particles, especially those with aerodynamic diameters with the 1–3 µm range, which is a standard size for spray-dried inhalable powders. While some degree of phagocytosis is inevitable, excessive clearance may hinder the delivery of active phages to their bacterial targets.

Additional immune challenges arise through recognition by host cells. Inhaled phages can be detected by alveolar epithelial cells and dendritic cells via pattern recognition receptors such as Toll-like receptor 9 (TLR9), which identifies unmethylated CpG motifs in phage DNA. TLR activation may trigger pro-inflammatory cytokines such as IL-6, IL-12, TNF-α, and IFN-γ, modifying local immune responses and altering phage pharmacokinetics [108]. Although purified phage preparations generally elicit mild responses, repeated exposure or residual bacterial lysates may provoke stronger immunogenicity, including the production of neutralizing IgA and IgG antibodies. These adaptative responses are particularly concerning in chronic pulmonary conditions such as CF or COPD, where baseline inflammation and compromised mucosal barriers exacerbate immune interactions [109,110].

Interestingly, certain phages exhibit immunomodulatory effects. For example, filamentous phages such as *Pf* from *Pseudomonas aeruginosa* can suppress oxidative bursts and promote M2 macrophage polarization. Other phages may inhibit phagocytosis or modulate interferon expression, revealing complex and phage-specific interactions with the host immune system [108]. Complementing these findings, recent pharmacokinetic studies have shown that neutrophils are not primary mediators of systemic phage clearance; rather, soluble factors such as complement proteins or extracellular enzymes are key players, indicating a distinction between systemic and pulmonary immune dynamics [111].

To enhance retention and therapeutic efficacy in the respiratory tract, formulation strategies have advanced beyond conventional sugar-based excipients to include polymeric systems that help to circumvent the lungs’ natural clearance mechanism. These include mucociliary transport, phagocytic intake by alveolar macrophages, enzyme degradation, and immune-mediated inactivation [112,113]. Mucoadhesive polymers such as chitosan, alginate, and carboxymethylcellulose can prolong the phage residence time in the airways by electrostatically interacting with mucins, enabling formulations to anchor within the mucus layer and resist mucociliary clearance [114]. This is especially important in CF patients, where thickened mucus impedes phage diffusion and bacterial access.

To reduce phagocytic uptake by alveolar macrophages, researchers have explored porous and low-density particle designs. Alveolar macrophages preferentially internalize dense particles with aerodynamic diameters of 1 and 3 µm. Using leucine composites or biodegradable polymers such as PLGA, it is possible to engineer particles with broader geometric sizes and lower densities, thereby avoiding macrophage detection and enabling deeper lung deposition [115,116,117]. Spray-drying and spray-freeze-drying techniques can be used to fabricate such porous structures while maintaining phage viability and aerosolization properties.

**Figure 9:**
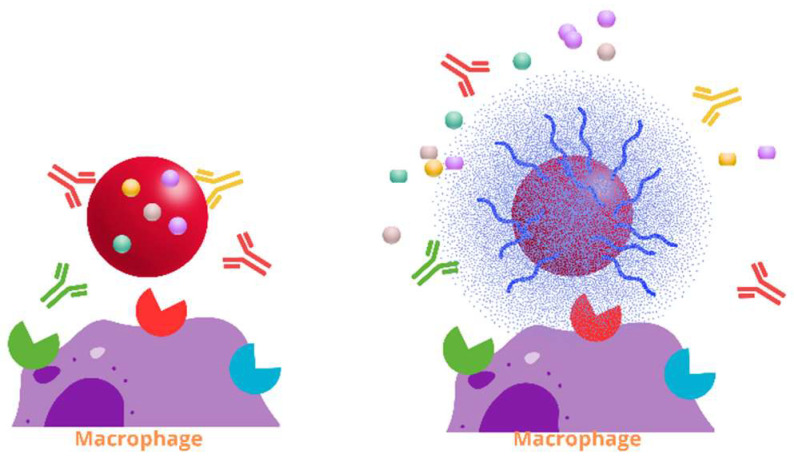
Comparison between conventional and PEGylated particles in their interactions with macrophages. While uncoated particles are readily opsonized and recognized by immune receptors, PEGylated particles exhibit a stealth effect, avoiding antibody binding and receptor-mediated uptake, thus prolonging the circulation or residence time in pulmonary tissue.

Encapsulation using biodegradable polymers such as PLGA or Eudragit^®^ also protects phages from immune detection and enzymatic degradation by pulmonary components, such as by lysozymes or surfactant proteins [118]. In chronic infections, these encapsulation methods enable sustained phage release, preserving their activity over time and shielding phages from neutralizing antibodies and immune effector molecules (Figure 9) [119].

Finally, nanoformulations, designed as biophysically engineered, multifunctional platforms, offer synergistic strategies to protect bacteriophages throughout manufacturing, storage, and delivery. These systems maintain phage bioactivity by substituting structural water, restricting molecular motion, reducing mechanical stresses, and providing protection from environmental assaults. By optimizing the deposition of viable phages at the infection site, they not only extend the shelf life of phage therapies under ambient conditions but also enhance their therapeutic efficacy. As such, nanoformulations are integral to the development of stable, effective, and clinically viable inhalable phage treatments.

In conclusion, polymeric carriers provide practical advantages such as improved mucosal adherence, immune evasion, and sustained release. While disaccharides and amino acids remain critical in stabilizing phages during drying and storage, the integration of polymer science into formulation design is essential to overcome respiratory barriers and improve clinical outcomes in inhalable phage therapy.

## 8. Regulatory and Quality Frameworks for Inhalable Phage Formulations

The path to the clinical translation of inhaled bacteriophage involves navigating complex and evolving regulatory frameworks. Although phages are recognized as biological products by both the FDA and the EMA, a unified global regulatory framework for them is still lacking. Phages present specific challenges due to their biological diversity, uniqueness, and, in some cases, personalized or adaptable nature [120].

In the United States, phage-based products fall under the jurisdiction of the FDA’s Center for Biologics Evaluation and Research, with natural phages evaluated as live biotherapeutics and genetically engineered ones possibly falling under the Office of Tissues and Advanced Therapies [121]. The EMA similarly supports phage therapy under Directive 2001/83/EC but lacks specific guidelines for phage therapy [122]. The absence of pharmacopeia standards for phage products is a major issue given that each developer has responsibility for independently verifying manufacturing procedures, quality control standards, and stability profiles in the absence of an approved monograph. Due to their physicochemical needs for pulmonary delivery, dry DPI systems present additional complexity, making this particularly crucial. The use of titer-based potency assays, genomic sequencing, and verified identity and sterility testing to characterize phages is increasingly recommended by regulatory organizations [123,124].

In accordance with the Good Manufacturing Practice guidelines, phage production must include standardized bacterial host banks, cleanroom propagation, scalable purification (e.g., TFF, chromatography), and reproducible formulation strategies. Quality control assays must evaluate crucial factors, including endotoxin concentrations, host-cell protein removal, genomic stability, and the absence of lysogenic features [125,126]. In addition to regulatory classification and manufacturing processes, strict quality control (QC) is required for phage-based therapies, particularly dry powder inhalers. QC must establish the safety, purity, identity, and potency of the final formulation using validated, regulatory-compliant methodologies. Environmental control is crucial during production; any process that involves exposure to open air must fulfill grade A cleanliness requirements. To confirm identity and genetic stability in host bacterial strains, molecular typing techniques such as multi-locus sequence typing, amplified fragment length polymorphism, repetitive PCR, and pulsed-field gel electrophoresis (PFGE) are used. The double-agar layer assay or time-kill assays are commonly used to assess functional potency, as they track lytic activity across target bacterial strains. These are supplemented by qPCR or ELISA for quantification, especially in phage mixtures where individual tracking is required. Importantly, next-generation sequencing is recommended to ensure phage identity and exclude lysogenic or virulence-associated genes, as lytic phages are preferable for therapeutic applications due to the lower risk of horizontal gene transfer.

Stability testing for dry powder phage formulations must follow the ICH Q1A(R2) guidelines, which include accelerated (40 °C/75% RH) and long-term (25 °C/60% RH) conditions. Chang, Kwok, Khanal, Morales, Kutter, Li, and Chan [63] and Leung, Parumasivam, Gao, Carrigy, Vehring, Finlay, Morales, Britton, Kutter, and Chan [102], found that phage survival and aerodynamic stability can be maintained for up to a year under optimal conditions. Despite the lack of approved phage DPI products, regulatory bodies are increasingly recognizing the importance of flexible, risk-based pathways.

## 9. Preclinical and Clinical Evidence of Inhalable Dry Powder Phages

Despite substantial preclinical advancements, clinical data on inhalable dry powder phage therapy remain limited. Most studies to date have been confined to in vitro evaluations of animal models, with only a few progressing to clinical translation. In a pivotal preclinical study, Chang et al. [127] demonstrated that dry powder formulations of bacteriophages targeting *Pseudomonas aeruginosa* retained their infectivity and effective lung deposition following inhalation. These powders also exhibited excellent aerosol performance and long-term stability when stored under low-humidity conditions for up to one year.

While no clinical trials have yet investigated dry powder phage formulations, several clinical programs have explored inhalable phage therapy delivered via nebulization, indicating the potential of this route in treating pulmonary infections due to its targeted deposition, ease administration, and formulation stability. Notably, Armata Pharmaceuticals has been at the forefront of clinical development, advancing two phage therapeutics: AP-PA02 and AP-SA02.

AP-PA02 is a nebulized phage cocktail designed to treat *Pseudomonas aeruginosa* infections in conditions such as non-cystic fibrosis bronchiectasis and cystic fibrosis (CF). In Phase 1b/2a (“SWARM-P.a.”) and Phase 2 (“Tail-wind”) clinical trials, AP-PA02 demonstrated a favorable safety profile. It also led to a reduction in *P. aeruginosa* sputum loads, with post hoc analyses reveling statistically significant CFU reductions and trends toward clinical improvement.

AP-SA02, administered intravenously, targets *Staphylococcus aureus*, including MRSA. In the Phase 1b/2a disarm trial, AP-SA02 yielded a significantly higher clinical response rate compared to the placebo (88% vs. 58% at day 12), with all treated patients achieving a full clinical response by the end of the research. These clinical findings demonstrate the potential effectiveness and safety of systemic and inhaled phage treatments, highlighting their value in treating infections that are resistant to several drugs. To take advantage of their specific benefits in terms of stability, mobility, and direct lung delivery, DPI phage systems must be advanced to clinical testing.

## 10. Challenges of Inhaled Phage Therapy

Inhaled phage therapy has emerged as a promising alternative for the treatment of pulmonary infections caused by MDR bacteria, particularly in diseases such as cystic fibrosis and bronchiectasis. Among the key pathogens implicated in these chronic infections is *Pseudomonas aeruginosa*, an opportunistic bacterium known for its robust biofilm formation and resistance to multiple antibiotic classes. In this context, inhaled bacteriophages represent a promising approach to overcoming the limitations of conventional therapies. However, clinical translation is hindered by several challenges, including phage stability during formulation, effective delivery within the pulmonary environment, and the design of optimized formulations that ensure both protection and targeted lung deposition.

Although the SD technique is widely used in the pharmaceutical industry, it subjects phages to severe desiccation and mechanical shear, which can significantly reduce their infectivity. Vandenheuvel, Singh, Vandersteegen, Klumpp, Lavigne, and Van den Mooter [11] demonstrated that excipients like trehalose act as protective agents during spray drying and subsequent storage. Similarly, Matinkhoo et al. [128] emphasized the importance of maintaining low drying temperatures to preserve phage viability in respirable microparticles. Leung, Parumasivam, Gao, Carter, Carrigy, Vehring, Finlay, Morales, Britton, Kutter, and Chan [77] further identified that the difference between the storage temperature and the glass transition temperature of the powder plays a critical role in preventing phage degradation during long-term storage.

Once inhaled, phages face a series of physiological barriers. Mucociliary can remove phages before they reach the bacterial target, while innate and adaptive immune responses may neutralize them prior to therapeutic action. In particular, *P. aeruginosa* is known to induce a strong inflammatory response in the lungs, potentially obstructing the effective delivery of phages. Agarwal, Johnson, Imhoff, Donlan, McCarty, and García [61] showed that polymeric microparticles can enhance pulmonary delivery and improve phage persistence in the lungs. Roach et al. [129] suggested that successful therapy may depend not only on the lytic activity of phages but also on their ability to synergize with the host immune system. Moreover, repeated administration may induce neutralizing immune responses, reducing treatment efficacy [130]. In murine models, certain phages have demonstrated the capacity to modulate inflammatory cytokine expression, thereby aiding tissue repair and mitigating lung damage [131].

Designing optimal inhalable formulations is critical. Beyond preserving phage viability during processing, the formulation must enable effective deposition in the lower respiratory tract, where chronic *P. aeruginosa* infections typically persist. Key aerodynamic properties of the dry powder, such as the particle size, morphology, and density, must be tightly controlled to ensure deep lung delivery. Chang, Wallin, Kutter, Morales, Britton, Li, and Chan [48] formulated powders incorporating mannitol, trehalose, and leucine, achieving high dispersibility and extended stability. Leung, Parumasivam, Gao, Carter, Carrigy, Vehring, Finlay, Morales, Britton, and Kutter [77] highlighted the importance of maintaining the conditions during storage to preserve phage integrity. Golshahi et al. [132] validated the in vitro effectiveness of these formulations when administered via dry powder inhalers, confirming their suitability for deep lung targeting.

Collectively, these studies underscore the complexity of translating inhaled phage therapy from the laboratory to clinical practice. Ensuring phage viability throughout production and storage, designing formulations that provide robust environmental protection and effective delivery, and accounting for immune system interactions and lung-specific barriers, particularly in the context of *P. aeruginosa* infections, are all critical. Ultimately, a multidisciplinary approach that integrates microbiology, pharmaceutical sciences, immunology, and aerosol technology is essential to advancing inhaled phage therapy into a reliable and effective clinical solution.

Recent studies have shown that inhaling bacteriophages can effectively target the lungs, especially when delivered through methods like dry powder formulations or nebulizers. For instance, research by Liu, Liu, Wang, Wan, Tian, Liu, Pang, and Wang [56] demonstrated that bacteriophages encapsulated in PLGA could not only reach the lungs of mice but also remain there, with detectable levels in lung tissue persisting up to 24 h after inhalation. Similarly, another study by Southard, Melton, Sandoval, Zaki, Williams III, and Cui [60] showed that a specific phage, D29, was successfully delivered to alveolar macrophages using a thin-film freezing method, demonstrating that, when it comes to how long these bacteriophages last in the lungs, they are usually cleared out within 48 to 72 h through mechanisms like mucociliary transport and phagocytosis. However, using encapsulation techniques can help them to remain in the lungs for a longer period of time. Interestingly, repeating the inhalation of these phages has not led to significant inflammation or damage to lung tissue in animal studies. Additionally, the immune response to these repeated doses seems to be relatively mild. While some antibodies, such as IgG or IgA, may develop, their production is often delayed or limited because of the use of polymer encapsulation or protective additives [119,129]. Overall, these findings are promising regarding the potential of using inhaled phages as a therapy, although we still need more long-term studies in humans to ensure that they are safe and effective.

## 11. Future Perspectives

Effective treatment is nevertheless hindered by the prevalence of diseases associated with biofilms and the global increase in MDR infections. Traditional antibiotics often struggle to penetrate biofilms and overcome bacterial resistance. When combined with antibiotics, mucolytic drugs (such as NAC or ambroxol), or β-lactamase inhibitors, phage-based combination therapies have synergistic effects, enabling them to break down biofilms, restore antibiotic sensitivity, and enhance clinical results [133]. For example, in murine models of ventilator-associated pneumonia caused by *Pseudomonas aeruginosa*, the combination of phages and meropenem led to a 4-log reduction in bacterial counts within four hours, significantly outperforming monotherapies [12]. In a practical context, in a recent multicenter propensity-matched study, aerosolized polymyxin B combined with inhalation led to greater microbial clearance (46.7% vs. 26.7%, *p* = 0.049) in carbapenem-resistant Gram-negative bacteria-infected stroke-associated pneumonia patients [134].

The inherent host specificity of phage therapy is one of its primary drawbacks. Since phages’ lytic activity depends on their ability to recognize specific bacterial surface receptors, which can vary not only between species but also within clinical isolates, they must be carefully matched to the infecting bacterial strain. This necessity makes standardized therapy more challenging and requires large phage libraries and rapid testing platforms. Furthermore, a patient’s respiratory microbiota composition can influence phage activity through the modification of the immune milieu, competition at binding sites, or the release of inhibitory factors. According to studies, phage pharmacodynamics and efficacy can be significantly impacted by differences in lung microbiomes between people with cystic fibrosis and healthy controls.

Despite promising results, the clinical translation of these strategies faces several obstacles. Optimizing dosing regimens and timing is essential to maximize synergy and avoid antagonistic interactions [41]. For example, transcription inhibitors such as rifampicin can hinder phage replication, diminishing therapeutic effectiveness when administered concurrently. Studies confirm that delivering phages before antibiotics enhances bacterial killing and biofilm disruption, leveraging phages’ ability to act on larger bacterial populations before antibiotic-induced reductions occur [135,136]. Moreover, variability in study designs, dosing regimens, routes of administration, and patient populations makes comparative analysis difficult. For inhalable dry powder formulations in particular, clinical data are virtually absent, with most studies focused on nebulized or intravenous delivery. Critical pharmacokinetic parameters—such as phage deposition, persistence in the lungs, and interactions with pulmonary immune defense—remain poorly characterized in humans.

## 12. Conclusions

The current challenges in phage therapy encompass regulatory, biological, and technical hurdles that complicate its clinical translation and widespread use. Despite their specificity and adaptability, phages are classified as biological products, and regulatory pathways—such as those enforced by the FDA—are still evolving to accommodate their unique nature. Phages require individualized selection due to their narrow host ranges, limiting accessibility and complicating the development of broad-spectrum phage products. Safety concerns mandate thorough genomic screening to exclude phages carrying genes encoding toxins or capable of horizontal gene transfer, as well as the assessment of endotoxin release from lysed bacteria. Additionally, the interaction between phages and the human immune system remains incompletely understood; pre-existing neutralizing antibodies and immune clearance can reduce phage efficacy, although innate immunity may synergize with phage therapy to eliminate bacterial subpopulations.

In summary, while phage therapy offers a promising solution for MDR bacterial infections, overcoming the current challenges is essential for its widespread clinical implementation. This includes establishing regulatory guidelines, developing standardized phage libraries, ensuring formulation stability, understanding immune interactions, and designing precise dosing strategies. A multidisciplinary approach involving microbiology, pharmacology, regulatory science, and immunology will be critical to unlocking the full potential of inhaled phase therapies.

## Figures and Tables

**Table 2:** Comparative summary of polymer-based phage nanocarriers for dry powder inhalation therapy.

Nanocarrier System	Particle Size	Phage Protection	Pulmonary Deposition Efficiency	Drying Method Compatibility	Reference
PLGA spheres	Geometric diameter: 10.67 µm Aerodynamic diameter: 4.62 µm	High (encapsulation preserves infectivity; minimal titer loss after freeze drying; stability ≥ 12 days at room temperature)	High (aerodynamic diameter in respirable range; lung retention confirmed after inhalation)	Vacuum freeze drying	[56]
PLGA spheres	Aerodynamic diameter: 3.3–3.8 µm	Lytic activity preserved	Cascade impaction results: geometric mean weight diameter: 6.6 µm	Stable immediately after freeze drying	[57]
Chitosan nanoparticles	Phage-loaded CS-NPs: 297 ± 18 nm	Encapsulation efficiency: ~97% Maintained infectivity under pH 3–12, at 25–80 °C	No direct lung model data	Not involved	[58]
Liposomes	PEV2: 301 ± 35.8 nm PEV40: 651 ± 14.3 nm	Titer reduction after nebulization	Viable respirable fraction (VRF) with vibrating mesh: ~70.3% (PEV2), 74.8% (PEV40) VRF with jet nebulizer: ~44% (PEV2), 28.2% (PEV40)	Not involved	[59]
PVP-K25 matrix powder	Mass median aerodynamic diameter: 2.84 µm	Preserved D29 viability post-TFF and during storage; active against intracellular M. smegmatis	Effective phage delivery to alveolar macrophages; targeted intracellular release	Thin-film freezing (TFF)	[60]

**Table 3:** Overview of drying techniques for phage powder formulations.

Drying Technique	Thermal Stress	Phage Recovery (%)	Particle Morphology	Aerodynamic Size	Cost/Scalability	References
Spray Drying	High (100–160 °C)	50–90%	Spherical, dense, or collapsed	1–5 µm	Low cost, high throughput	[24,63]
Lyophilization	Low (frozen)	>90% (with stabilizers)	Irregular, requires milling	3–15 µm (post-milling)	High cost, slow	[57]
Spray Freeze Drying	Very low (−100 to −130 °C)	60–85%	Porous, spherical	2–6 µm	Medium–high, moderate scale-up	[64,65]
Thin-Film Freeze Drying	Minimal (−40 °C films)	>90%	Fragile, porous films	1–5 µm (after milling)	High, emerging technique	[60]

**Table 4:** Overview of dry powder bacteriophage formulations.

Phage Studied	Powder Manufacturing Process	Excipients	Previous Phage Encapsulation	Notable Results	Reference
Phage cocktail (against *Pseudomonas aeruginosa*)	Freeze drying or lyophilization	Lactose	Yes, into PLGA microparticles	The study, conducted in a murine model, resulted in a significant reduction in the bacterial load in the lungs, indicating the potential efficacy of the treatment.	[61]
Phage PEV20 (against *Pseudomonas aeruginosa*) and ciprofloxacin	Spray drying	Leucine, with and without lactose	No	The formulations maintained antimicrobial synergy.	[6]
Phage cocktail PEV2, PEV1 and PEV20 (against *Pseudomonas aeruginosa*)	Spray drying	Lactose and leucine	No	The formulation of 80:20% lactose–leucine retained phage viability and achieved an FPF of up to 45%. Its in vitro efficacy against MDR strains was demonstrated.	[73]
Phage PEV2 (against *Pseudomonas aeruginosa*)	Spray drying	HSA–lactose	No	First study using HSA as an excipient. The formulation HSA–lactose 60:40% w/w demonstrated favorable results with lower titer loss (<log) and FPF > 50%. Future investigations need to be performed.	[71]
Mycobacteriophage (against *Mycobacterium tuberculosis*)	Freeze drying or lyophilization	Trehalose, leucine, and cyclodextrin	No	Although the formulation exhibited antibacterial activity in vitro and achieved FPF > 50%, it failed to produce a significant antibacterial effect in vivo. These findings underscore the challenges associated with translating in vitro efficacy into in vivo therapeutic outcomes.	[64]

## Data Availability

Dataset available on request from the authors.

## Abbreviations

The following abbreviations are used in this manuscript:

Abbreviation	Meaning
CFU	Colony-Forming Unit
PFU	Plaque-Forming Unit
RH	Relative Humidity
Tg	Glass Transition Temperature
SEM	Scanning Electron Microscopy
SFD	Spray Freeze Drying
COPD	Chronic Obstructive Pulmonary Disease
TEM	Transmission Electron Microscopy
TGA	Thermogravimetric Analysis
DPI	Dry Powder Inhaler
MDR	Multidrug Resistance
M. smegmatis	Mycobacterium smegmatis
PVA	Polyvinyl Alcohol
R-M	Restriction–Modification
CF	Cystic Fibrosis
PEV	Pseudomonas Phage
REases	Restriction Endonucleases
CRISPR-Cas	Clustered Regularly Interspaced Short Palindromic Repeats
CBASS	Cyclic Oligonucleotide-Based Anti-Phage Signaling System
pMDIs	Pressurized Metered Dose Inhalers
FPF	Fine Particle Fraction
VRF	Viable Respirable Fraction
TFF	Thin-Film Freezing
PVP	Polyvinylpyrrolidone
PLGA	Poly(Lactic-Co-Glycolic Acid)
TLR	Toll-Like Receptor
QC	Quality Control
